# Evaluate prognostic accuracy of SOFA component score for mortality among adults with sepsis by machine learning method

**DOI:** 10.1186/s12879-023-08045-x

**Published:** 2023-02-06

**Authors:** Xiaobin Pan, Jinbao Xie, Lihui Zhang, Xincai Wang, Shujuan Zhang, Yingfeng Zhuang, Xingsheng Lin, Songjing Shi, Songchang Shi, Wei Lin

**Affiliations:** 1 Department of Critical Care Medicine, Shengli Clinical Medical College of Fujian Medical University, Fujian Provincial Hospital South Branch, Fujian Provincial Jinshan Hospital, Fujian Provincial Hospital, Fuzhou, China; 2 Department of Thoracic Surgery, First Affiliated Hospital of Fujian Medical University, Fuzhou, China; 3 Department of Critical Care Medicine, Shengli Clinical Medical College of Fujian Medical University, Fujian Provincial Hospital, Fuzhou, China; 4 Department of Endocrinology, Shengli Clinical Medical College of Fujian Medical University, Fujian Provincial Hospital, Fuzhou, China

**Keywords:** Sepsis, Hospital mortality, Intensive care unit, Machine learning, Sequential organ failure assessment

## Abstract

**Introduction:**

Sepsis has the characteristics of high incidence, high mortality of ICU patients. Early assessment of disease severity and risk stratification of death in patients with sepsis, and further targeted intervention are very important. The purpose of this study was to develop machine learning models based on sequential organ failure assessment (SOFA) components to early predict in-hospital mortality in ICU patients with sepsis and evaluate model performance.

**Methods:**

Patients admitted to ICU with sepsis diagnosis were extracted from MIMIC-IV database for retrospective analysis, and were randomly divided into training set and test set in accordance with 2:1. Six variables were included in this study, all of which were from the scores of 6 organ systems in SOFA score. The machine learning model was trained in the training set and evaluated in the validation set. Six machine learning methods including linear regression analysis, least absolute shrinkage and selection operator (LASSO), Logistic regression analysis (LR), Gaussian Naive Bayes (GNB) and support vector machines (SVM) were used to construct the death risk prediction models, and the accuracy, area under the receiver operating characteristic curve (AUROC), Decision Curve Analysis (DCA) and K-fold cross-validation were used to evaluate the prediction performance of developed models.

**Result:**

A total of 23,889 patients with sepsis were enrolled, of whom 3659 died in hospital. Three feature variables including renal system score, central nervous system score and cardio vascular system score were used to establish prediction models. The accuracy of the LR, GNB, SVM were 0.851, 0.844 and 0.862, respectively, which were better than linear regression analysis (0.123) and LASSO (0.130). The AUROCs of LR, GNB and SVM were 0.76, 0.76 and 0.67, respectively. K-fold cross validation showed that the average AUROCs of LR, GNB and SVM were 0.757 ± 0.005, 0.762 ± 0.006, 0.630 ± 0.013, respectively. For the probability threshold of 5–50%, LY and GNB models both showed positive net benefits.

**Conclusion:**

The two machine learning-based models (LR and GNB models) based on SOFA components can be used to predict in-hospital mortality of septic patients admitted to ICU.

**Supplementary Information:**

The online version contains supplementary material available at 10.1186/s12879-023-08045-x.

## Introduction

Sepsis is a life-threatening failure due to dysregulated immune response to infections and an important global health problem, and is particularly common in intensive care unit (ICU) [1–3]. The latest global sepsis incidence and mortality estimates report shows that estimated 48.9 million sepsis cases and 11.0 million sepsis-related deaths occur each year [3]. It is a great burden to the patient’s family and society as a whole. Early risk stratification for sepsis patients and targeted treatment measures are of great significance to reduce mortality and improve prognosis.

At present, sequential organ failure assessment (SOFA), Early warning scores (EWS) and other scores are used in clinical risk stratification for patients with sepsis [4, 5], but is not without limitations. As we know, SOFA score is one of the main criteria for sepsis 3.0 diagnosis, previous studies have evaluated the predictive value of SOFA score in patients with sepsis, however it has shown varying performance in predicting short-term mortality [6–8], the generalization ability of prediction models were limited. A retrospective study evaluated the prognostic values of SOFA score in sepsis patients, The area under the receiver operating characteristic curve (AUROC) of short-time mortality was 0.661, which showed general predictive ability [9]. SOFA score also showed moderate predictive performance (AUROC 0.61) in a recent study [10]. The following reason may contribute to this result. SOFA score consists of six organ system scores, and each set to 0–4 points, previous studies simply used the SOFA score to death risk prediction, meaning simply added up the scores of the six organ systems, and didn’t take into account that the various organ systems may be have different effects on the mortality risk of septic patients, this may reduce the predictive power of the model.

In recent years, machine learning has been used more and more widely in the field of medicine and is regarded as a very effective method [11–13], especially in developing predictive models and identifying important factors [14]. What’s more, most existing studies show that the performance of machine learning models for predicting mortality risk in septic patients were improved over traditional clinical scoring systems [15, 16]. A recently published study developed a prognostic machine learning model based on SOFA to predict short-term mortality risk for septic patients, and the AUROC was 0.742, which performed better than traditional risk prediction models [17]. Yet, we want to get more accurate models.

In this study, we took into account the different weight of influence of organ system disorders on the prognosis of sepsis patients, and developed machine learning models based on component scores of SOFA to early predict in-hospital mortality in ICU patients with sepsis, and to evaluate the performance of the models.

## Methods

### Data sources

**Fig.1:**
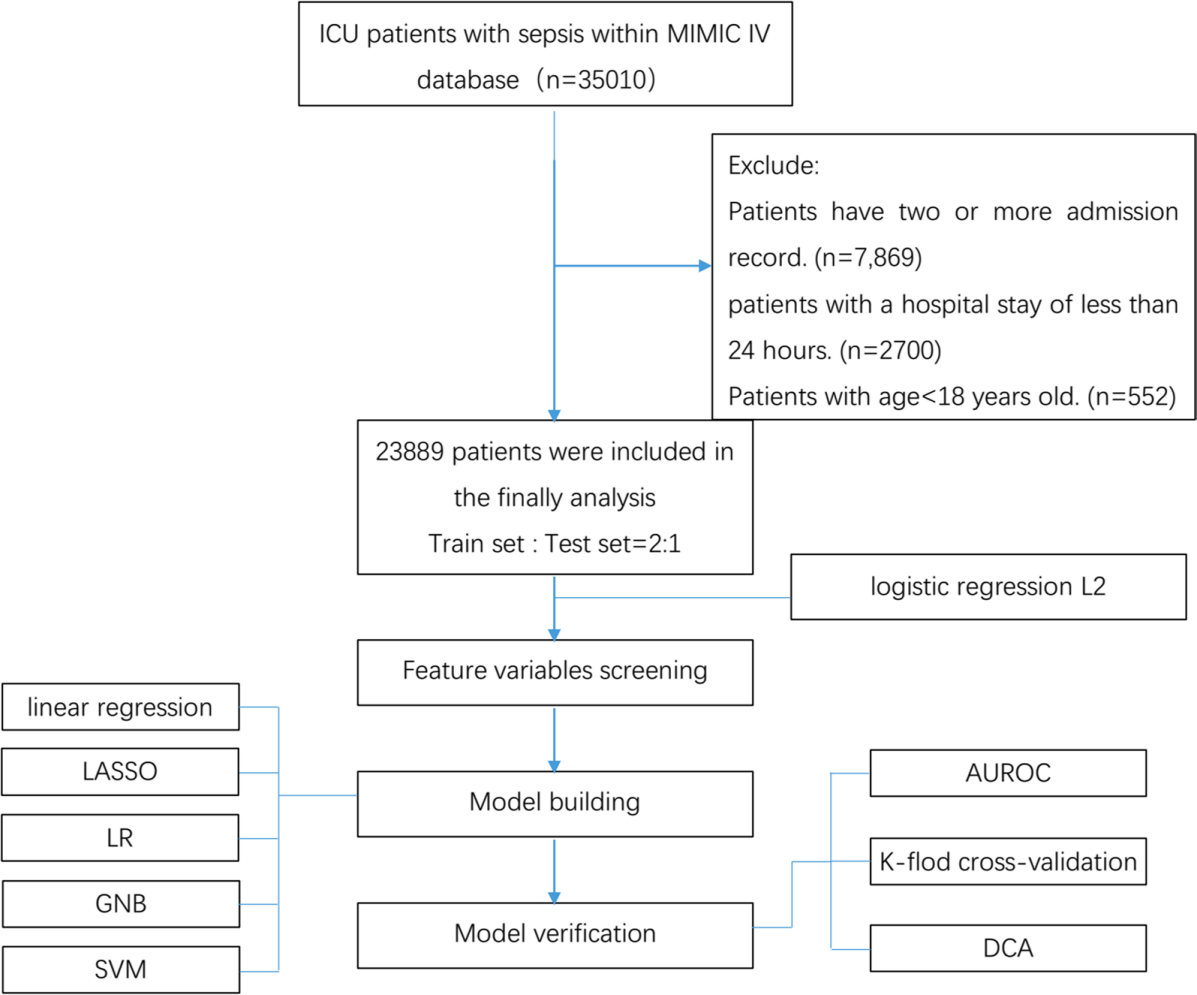
Flowchart of the research process. *LASSO* least absolute shrinkage and selection operator, *LR* logistic regression analysis, *GNB* Gaussian Naive Bayes, *SVM* support vector machines, *AUROC* area under the receiver operating characteristic curve, *DCA* decision curve analysis

This was a retrospective cohort study. We extracted data from mimic-IV (Version 1.0) database [18]. The MIMIC IV database is publicly available and contains over 40,000 ICU patients who admitted to the ICUs of Beth Israel Deaconess Medical Center between 2008 and 2019. The database provides data on the entire hospitalization process of patients admitted to ICU, including demographic data, vital signs, laboratory results, surgery, medication, monitoring records, imaging reports, and deaths, and provides data support for many analytical studies. It is approved by the Massachusetts Institute of Technology Institutional Review Boards. All the patients of the database are de-identified for privacy protection and informed consent are waived by Fujian Provincial Hospital Ethics Committee. The use of the data was authorized by the Massachusetts Institute of Technology Affiliates. Author Xiaobin Pan obtained database access authorization (certification number: 36845846) through related study and assessment, and completed data extraction through SQL language. The study followed the principles of the Declaration of Helsinki. Flowchart of the research process were showed in Fig. 1.

### Patients and definitions

Patients admitted to ICU with Sepsis3.0 diagnosis were extracted. The diagnostic criteria for Sepsis are suspected infection and SOFA score ≥ 2 [2, 19]. Patients younger than 18 years of age and patients with a hospital stay of less than 24 h were excluded. If the patient is hospitalized repeatedly, the information of the first hospitalization is taken.

### Data extraction

The highest SOFA score and 6 SOFA component scores (including respiratory system score, coagulation system, liver system score, renal system score, cardio vascular system score, and nervous system score) in SOFA score were collected on the first 24 h of admission. The six organ function scores were set to 0–4. Baseline characteristics and outcome variable were also collected at admission to the ICU including age, sex, hospital mortality or not. All of the above data were extracted within 24 h of admission.

### Statistical analysis

All data in this study were analyzed by R language software (version 3.8.8), and the missing data were filled by KNN Imputer. Standard deviation (SD) or medians with interquartile ranges (IQRs) were used to describe the patient’s characteristic data. Firstly, the extracted data were randomly divided into training set and test set in accordance with 2:1, the StandardScaler was used to standardize the data, which is a preprocessing module. Secondly, Feature variables were selected from 6 organ system scores in the SOFA score for model construction. Our study used SelectFromModel [20] as a transformer to be an estimator that assigns importance to features. If the importance of the feature values is lower than the threshold parameter set, SelectFromModel considers these features to be unimportant and removes them. This study employed L2-based feature selection (penalty = “L2,” c = 0.05). Subsequently, the importance of the feature values above the threshold was retained. This method can be used for risk factor screening and high-dimensional data analysis [21, 22]. Finally, six machine learning methods including linear regression analysis, least absolute shrinkage and selection operator (LASSO), Logistic regression analysis (LR), Gaussian Naive Bayes (GNB) and support vector machines (SVM) were used to construct the death risk prediction model, and the accuracy, area under the receiver operating characteristic curve (AUROC), Decision Curve Analysis (DCA) and K-fold cross-validation were used to evaluate the prediction performance of developed models.

## Results

### Baseline characteristic

In our study, a total of 23,889 patients with sepsis were enrolled, of whom 3659 died in hospital. The mean age of all participants was 65.06 ± 16.36 years, and the proportion of male patients was 57.77%. The baseline observation, comorbidities, blood results, SOFA score, length of stay in hospital and ICU, and outcome data were showed in Additional file 1: Table S1. Six variables were included in this study, all of which were from the scores of 6 organ systems (including respiratory system, coagulation system, liver system, renal system, cardio vascular system, and cerebral nervous system) in SOFA score. The proportion of variables were coagulation system (0.2%), liver system score (18.2%), renal system score (0.01%), cardio vascular system score (0.1%), and nervous system score (0.1%), respiratory system score (19.4%), respectively. The data were randomly divided into the train set (16,005 cases) and the test set (7884 cases) in 2:1 order. The baseline characteristics of the study variables are shown in Table 1. The data of training set were used for model construction. Flowchart of the research process were showed in Fig. 1.

**Table 1:** Baseline characteristics of 6 SOFA component scores

Variables	All patients	Train set	Test set
	(n = 23,889)	(n = 16,005)	(n = 7,884)
Respiration system score	2.04 ± 1.14	2.05 ± 1.14	2.03 ± 1.15
Coagulation system score	0 (0–1)	0 (0–1)	0 (0–1)
Liver system score	0 (0–1)	0 (0–1)	0 (0–1)
Renal system score	1 (0–2)	1 (0–2)	1 (0–2)
Cardio vascular system score	1.64 ± 1.33	1.63 ± 1.33	1.66 ± 1.33
Cerebral nervous system score	1.43 ± 1.33	1.43 ± 1.33	1.42 ± 1.32
Hospital mortality	3659 (15.3%)	2440 (15.2)	1219 (15.5%)

Data are showed as the mean ± SD or median (interquartile range)

### Feature variable

**Fig. 2:**
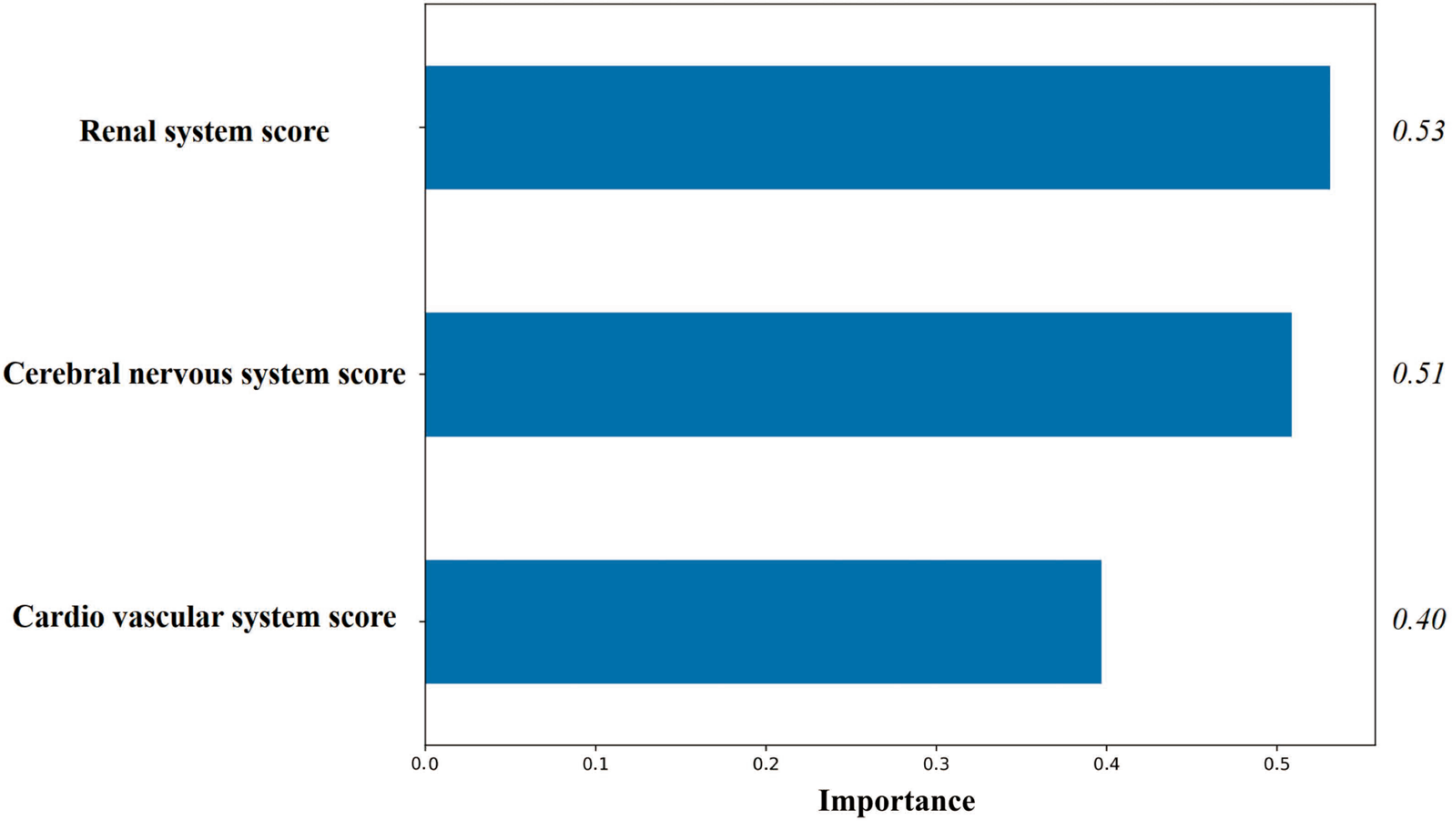
Importance of Feature variables screened by logistic regression L2 regularization

Three feature variables including renal system score, central nervous system score and cardio vascular system score were screened by logistic regression L2 regularization (Fig. 2).

### Prediction model

Three screened feature variables were used to construct six prediction models of in-hospital mortality of patients with sepsis, including linear regression analysis, LASSO, LR, GNB, SVM, The accuracy of training set and test set were 0.123 and 0.123, 0.123 and 0.130, 0.851 and 0.851, 0.854 and 0.844, 0.862 and 0.862, respectively. Obviously, LR, GNB, SVM show good accuracy.

### Model validation and clinical use

**Fig. 3:**
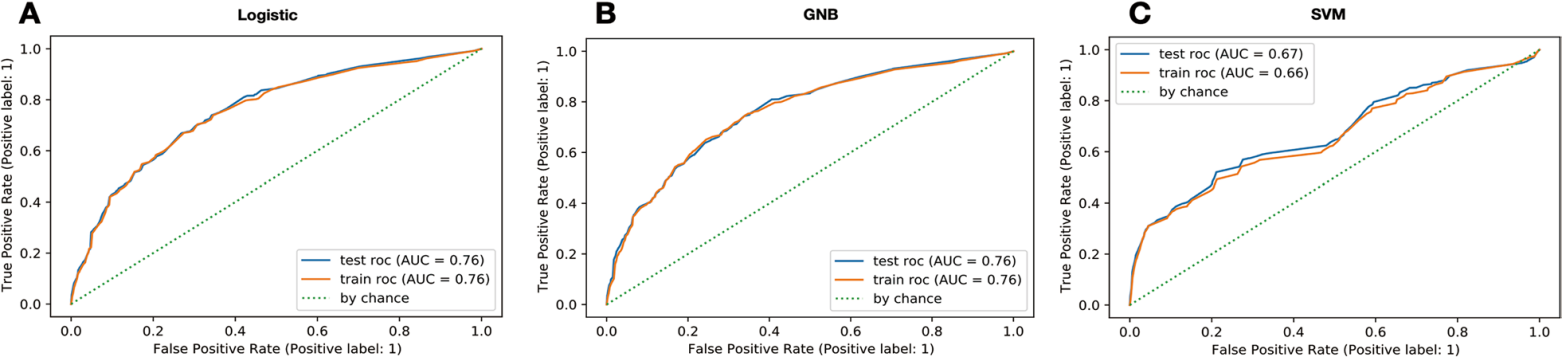
Receiver operating characteristic (ROC) curves of LR, GNB and SVM models in predicting hospital mortality. **A** ROC curves of LR; **B** ROC curves of GNB; **C** ROC curves of GNB; The y-axis represents the TPR of the risk prediction, the x-axis represents the FPR of the risk prediction. The ROC curve computed in training (red solid line) and testing (blue solid line) in each graph. *LR* logistic regression analysis, *GNB* Gaussian Naive Bayes, *SVM* support vector machines

**Fig. 4:**
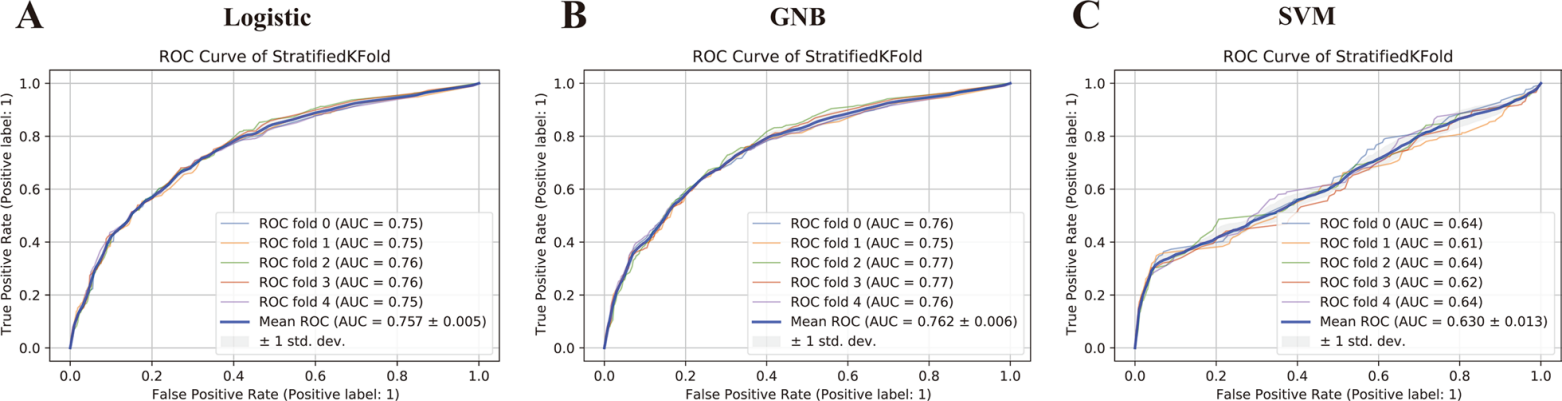
Receiver operating characteristic (ROC) curves of LR, GNB and SVM models in predicting hospital mortality by K-fold cross-validation. The fold value of k is taken as 5 in this study. **A** ROC curves of LR; **B** ROC curves of GNB; **C** ROC curves of GNB; The y-axis represents the TPR of the risk prediction, the x-axis represents the FPR of the risk prediction. fivefold cross-validation divides the dataset into five parts. Four of them is used for training and one of them is used for testing. This process is repeated five times until all data are used for testing and only once. We integrated the results of five validations, took the average value, and expressed it by mean ROC (blue solid line in each graph). *LR* logistic regression analysis, *GNB* Gaussian Naive Bayes, *SVM* support vector machines

**Fig. 5:**
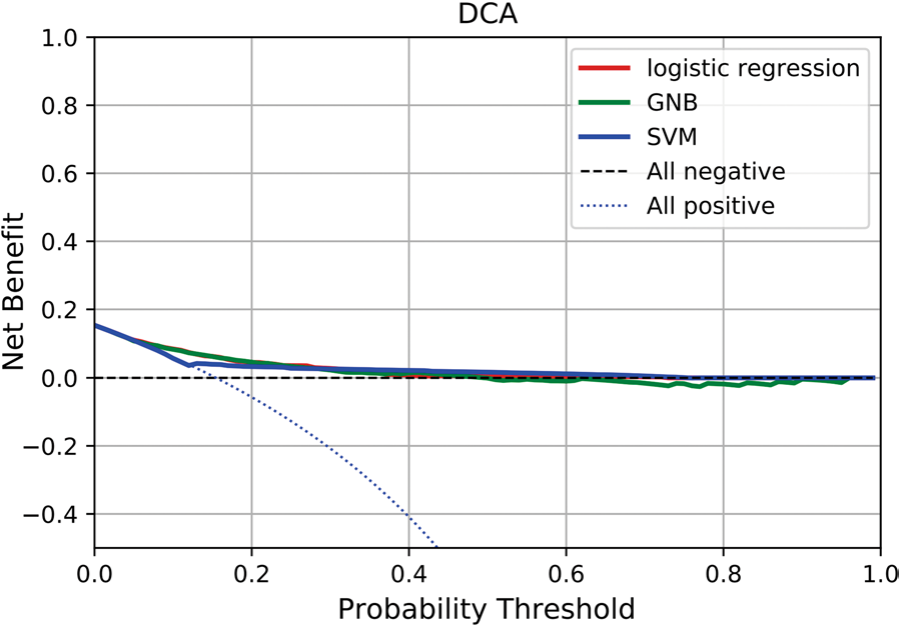
Decision curve analysis showing the clinical application value of LR, GNB and SVM models in predicting hospital mortality. The y-axis represents the net benefit; The x-axis represents the threshold probability. The blue dotted line represents the net benefit of providing all participants similar treatment, assuming that all would die. The black dotted line represents the assumption that all patients survive. The Red solid, green solid, and blue solid lines represent the net benefit of predicting the risk of in-hospital death according to LR, GNB, and SVM models, respectively. *LR* logistic regression analysis, *GNB* Gaussian Naive Bayes, *SVM* support vector machines

The areas under ROC curve (AUROC) of LR, GNB and SVM are 0.76, 0.76, 0.66, respectively, and 0.76, 0.76, 0.67 in the test set, which all perform well. LR and GNB had larger AUROC (Fig. 3). K-fold cross validation showed that the average AUROCs of LR, GNB and SVM were 0.757 ± 0.005, 0.762 ± 0.006, 0.630 ± 0.013, respectively. As shown in Fig. 4 the receiver operating characteristic curves of LR model and GNB model were similar. They show better effectiveness than SVM model, and have good stability and generalization ability. The DCA results of the three models (LR, GNB, and SVM) are shown in Fig. 5. For the probability threshold of 5–50%, LR model and GNB model both showed positive net benefits. The two models showed similar performance and were superior to SVM model, indicating higher clinical application value. To sum up, the performance of LR model based on SOFA score is similar to that of GNB model, which has moderate prediction ability and is superior to SVM model.

## Discussion

Our results suggest that machine learning based on SOFA components can be used to predict the risk of death in sepsis patients and has predictive value. Five machine learning models were built in our study, and the accuracy of LR, GNB and SVM was significantly better than the other two models. LR and GNB models had similar performance and were better than SVM. We further conducted k-fold cross validation and showed that LR and GNB models both had similar AUROC in each cross validation, indicating good generalization ability. In the model, SOFA components were selected as the predictive factors of each organ system function, which could be obtained quickly and easily in the hospital. Renal system score, central nervous system score and circulatory system score were the three feature variables of machine learning models in our study. To our knowledge, this was the first time that SOFA score was subdivided into 6 organ scores and machine learning was used to establish a prediction model for the assessment of mortality risk of sepsis patients, providing a more simple and effective prognostic tool for sepsis patients.

Sepsis was characterized by high morbidity and mortality [23], Early and accurate risk assessment of death is particularly important, and the establishment of prediction model is helpful for clinical decision support. GNB classification has been applied in many researches of classification problem. Its algorithm is simple to calculate, the calculation speed is relatively fast, and the classification results obtained are relatively high precision. Moreover, it can also show a good effect when the data volume is large. LR is a widely used linear classification model, which has the characteristics of strong interpretation and good predictive performance. Our prediction models (LR: AUC 0.76, GNB: AUC 0.76) were more accurate in stratifying mortality risk in sepsis patients than the previous SOFA score-based prediction model whose research object were also from the MIMIC-IV database (AUC 0.61) [10] and other previous SOFA score-based prediction models including (AUC 0.612–0.752) [6–10, 17, 19]^.^ The main possible reasons are as follows: Firstly. The traditional prediction models didn't consider the interaction among six organ system variables. Machine learning can obtain unknown dependencies from existing data sets and analyze and process new data sets with the learned dependencies. Secondly, The SOFA score included six organ system scores. It could be seen that different organ systems have different weights for predicting the risk of death. Previous studies all adopted the total SOFA score, which simply added up the scores of various organ systems and could not reflect their weight, which may affect the accuracy of the prediction model.

The three feature variables in our study were renal system score, central nervous system score, and cardio vascular system in SOFA score. Previous studies have shown that the top three organ systems with the greatest impact on prognosis in SOFA score were the cerebral nervous system, renal system and circulatory system, which was consistent with this study [24]. Among the three feature variables, renal system had the highest weight. Acute kidney injury (AKI) was the most common complication of sepsis, and the more severe the sepsis patients were, the higher the incidence of AKI was [25]. Acute kidney injury occurs earlier in sepsis patients, usually within 24 h of admission to ICU [26, 27], which can significantly prolong the length of hospital stay and increase mortality of patients with sepsis, and is an independent risk factor for death of patients with sepsis [28–30]. Previous studies have shown that the clinical prognosis of patients with septic AKI was more likely to be poor, and the nosocomial mortality was as high as 30–50%, and the more severe AKI grade was, the higher the mortality was [31–33]. A retrospective cohort study showed that the mortality rate of sepsis patients with acute renal failure (56.7%) was significantly higher than that of sepsis patients without acute renal failure (22.6%) [34]. The reason may be related to the pathophysiology of AKI caused by sepsis, including inflammation, hemodynamic changes, microvascular dysfunction, oxidative stress, and metabolic reorganization, which are related to the severity of sepsis and poor outcome [35]. In addition, the variables included in our study were only the composition of SOFA score, without stratified analysis of age and comorbidities, etc. Therefore, confounding factors could not be excluded, leading to that renal system had the higher weight than hemodynamic and CNS dysfunction. Septic shock is the most severe forms of sepsis, in recent years for the treatment of septic shock in improving unceasingly, but the mortality of septic shock patients is still as high as 35–40% [36], which is significantly higher than patients without septic shock, suggesting that cardio vascular system dysfunction plays a key role in promoting the poor prognosis of sepsis. In the SOFA score, the cardio vascular system score is mainly based on mean arterial pressure (MAP) and administration of vasopressors required. MAP is the main hemodynamic variable reflecting organ perfusion driving pressure. Lee et al. found that MAP was an independent risk factor for death in septic patients, and the MAP was lower than 65 mmHg means higher mortality [37]. Increased require of vasopressors in patients with septic shock was positively associated with increased mortality in a multicenter prospective cohort study [38]. Burstein et al. pointed out that the mortality rate of patients with severe sepsis requiring high doses of vasopressors was very high and increased as the demand for vasopressors increased [39]. In previous studies, it has been recognized that the central nervous system is one of the organs first involved in sepsis and also one of the organs most frequently involved in sepsis [40, 41]. In the SOFA score, Nervous system dysfunction is based on the Glasgow Coma Score. Previous studies had shown that the severity of brain dysfunction assessed by GCS score was positively correlated with the nosocomial mortality of patients with sepsis, and GCS was an independent risk factor for death of patients with sepsis in ICU [42–44], which was consistent with our study.

The merits of this study are as follows: Firstly, the data in this study came from MIMIC IV public database, and machine learning method was adopted to hand missing values and establish mortality risk prediction model, which was more consistent with objective facts. Secondly, we further subdivided SOFA score into six organ system scores, and selected feature variables by machine learning to further build the prediction model, fully considering the different weight of each organ system in SOFA score, and improved the prediction ability of SOFA score based prediction model.

However, there are limitations in this study: firstly, the validation of models were realized through internal verification of the test set, which needs to be verified by external validation in future studies; Secondly, the variables adopted in our prediction model are only the content of SOFA score. Since the purpose of our study is to better use SOFA score to predict the risk of death in patients with sepsis, our study did not consider other risk factors for death and we didn’t do stratified analysis of source of infection, severity of the subject and comorbidities, etc. This makes the model does not achieve the ideal predictive ability. Next, we will further combine other clinical indicators and biological markers to build more accurate models based on this study. In addition, this paper observes the outcome without reflecting the effect of time on the outcome. What’s more, we excluded patients discharged from hospital within 24 h in order to obtain more complete case data and scores of patients, which had a higher mortality rate (18.7%), which may have skewed the results. Finally, the data in this paper came from MIMIC IV public database, which was a retrospective cohort study and were collected from relatively long periods (2008–2019), which might be risk of bias due to change of standard of care/protocol of sepsis. Patients suspected of infection could only be extracted from the database system diagnosis, which could not fully reflect the real situation.

## Conclusion

The two machine learning-based models (LR and GNB models) based on SOFA components can be used to predict in-hospital mortality of septic patients admitted to ICU, and help clinicians to conduct targeted management of patients with different mortality risk levels in an early and timely manner.

## Supplementary Information

**Additional file 1.** Baseline clinical data of the included septic patients.

## Data Availability

The datasets used and/or analyzed during the current study are available from the corresponding author on reasonable request.
