# Supplementary material for: Evaluate prognostic accuracy of SOFA component score for mortality among adults with sepsis by machine learning method
Source: BMC Infect Dis. 2023 Feb 6;23:76. doi: 10.1186/s12879-023-08045-x (PMC9903420; doi:10.1186/s12879-023-08045-x)
Supplement: Supplementary file 1 — Additional file 1. Baseline clinical data of the included septic patients. [file 12879_2023_8045_MOESM1_ESM.docx]

**Supplementary Table 1 Baseline clinical data of the included septic patients**

|  | Survivors  (n = 20230) | Non-survivors  (n = 3659) |
| --- | --- | --- |
| Age (years) | 64.39 ± 16.42 | 68.76 ± 15.48 |
| Female, n (%) | 8450 (41.8%) | 1639(19.4%) |
| Temperature (◦C) | 37.53 ± 0.77 | 37.37 ± 1.08 |
| MAP (mmHg) | 57.63 ± 12.51 | 52.01 ± 15.80 |
| HR (beats/min) | 104.61 ± 20.24 | 112.58 ± 23.51 |
| RR (/min) | 28.04 ± 6.44 | 30.41 ± 6.98 |
| Charlson comorbidity score | 5.56 ± 2.87 | 7.01 ± 2.97 |
| Comorbidities, n (%) |  |  |
| MI | 3266 (16.14%) | 762(20.83%) |
| CHF | 5501(27.19%) | 1246 (34.05 %) |
| Diabetes | 2613(12.92%) | 1,061 (29.00 %) |
| COPD | 5115(25.28%) | 1,011(27.63%) |
| Liver disease | 2657(13.13%) | 934(25.53%) |
| Renal disease | 4128(20.41%) | 950(25.96%) |
| Cerebral vascular disease | 2,613(12.92%) | 730(19.95%) |
| Peripheral vascular disease | 2,364(11.69%) | 488(13.34%) |
| Laboratory tests |  |  |
| WBC count (*10^9^/l) | 15.16±11.00 | 18.11±19.10 |
| Hemoglobin (g/dl) | 9.95±2.12 | 9.68±2.34 |
| Hematocrit(%) | 29.87±6.31 | 29.69±7.03 |
| Platelet count (*10^9^/l) | 178.46±100.02 | 170.74±115.54 |
| BUN (mg/dl) | 28.46±22.71 | 41.39±29.20 |
| Creatinine(mg/dl) | 1.10(0.80-1.60) | 1.60(1.00-2.60) |
| Glucose (mg/dl) | 167.76±99.42 | 201.84±124.47 |
| Sodium(mmol/l) | 139.94±4.91 | 140.47±6.87 |
| Chloride(mmol/l) | 106.74±6.29 | 105.93±8.14 |
| Potassium(mmol/l) | 3.90±0.56 | 3.95 ±0.72 |
| PT(s) | 17.12±10.47 | 22.91± 17.84 |
| Oxygenation Index | 195.54±113.83 | 148.92 ±111.14 |
| Lactate(mmol/l) | 2.82±2.21 | 5.04±4.53 |
| SOFA score | 5.87±3.28 | 9.63±4.38 |
| Other outcomes, n (%) |  |  |
| Length of ICU stay (days) | 5.28±6.52 | 7.06±7.27 |
| Length of hospital stay (days) | 12.82±12.65 | 12.02±17.40 |

Abbreviation: BUN, blood urea nitrogen; HR, heart rate; MAP, mean arterial pressure; RR, respiratory rate; COPD, chronic obstructive pulmonary disease; CHF, congestive heart failure; MI, myocardial infarction; WBC, white blood cell; ICU, intensive care unit.
